# Development and Testing of the Aftercare Problem List, a Burn Aftercare Screening Instrument

**DOI:** 10.3390/ebj5020008

**Published:** 2024-03-29

**Authors:** Nancy E. E. Van Loey, Elise Boersma-van Dam, Anita Boekelaar, Anneke van de Steenoven, Alette E. E. de Jong, Helma W. C. Hofland

**Affiliations:** 1 Urban Vitality, Centre of Expertise, Faculty of Health, Amsterdam University of Applied Sciences, 1105 BD Amsterdam, The Netherlands; 2 Clinical Psychology, Faculty of Social Sciences, Utrecht University, 3584 CS Utrecht, The Netherlands; m.e.boersma-vandam@uu.nl; 3 Burn Centre, Red Cross Hospital, 1942 LE Beverwijk, The Netherlandsaeedejong@rkz.nl (A.E.E.d.J.); 4 Burn Centre, Maasstad Hospital, 3079 DZ Rotterdam, The Netherlandshoflandh@maasstadziekenhuis.nl (H.W.C.H.)

**Keywords:** burns, screening, scars, body image, stigmatization, positive coping, intimacy, relations, functional problems, psychological problems

## Abstract

A growing interest in person-centered care from a biopsychosocial perspective has led to increased attention to structural screening. The aim of this study was to develop an easy-to-comprehend screening instrument using single items to identify a broad range of health-related problems in adult burn survivors. This study builds on earlier work regarding content generation. Focus groups and expert meetings with healthcare providers informed content refinement, resulting in the Aftercare Problem List (APL). The instrument consists of 43 items divided into nine health domains: scars, daily life functioning, scars treatment, body perceptions, stigmatization, intimacy, mental health, relationships, financial concerns, and a positive coping domain. The APL also includes a Distress Thermometer and a question inquiring about preference to discuss the results with a healthcare provider. Subsequently, the APL was completed by 102 outpatients. To test face validity, a linear regression analysis showed that problems in three health domains, i.e., scars, mental health, and body perceptions, were significantly related to higher distress. Qualitative results revealed that a minority found the items difficult which led to further adjustment of the wording and the addition of illustrations. In summation, this study subscribes to the validity of using single items to screen for burn-related problems.

## 1. Introduction

The health and well-being of burn survivors can be affected for a prolonged period by a wide variety of physical, psychological, and social problems. Burns can result in hypertrophic scarring [1] and functional limitations [2] and typically cause problems such as pain and itch [3,4,5]. Well-established psychological problems comprise posttraumatic stress and depressive symptoms, anxiety, and body image concerns [6,7,8,9], often complicated by social problems [10], stigmatization, or vocational problems [11,12,13]. Furthermore, fatigue and sleep problems were identified as enduring problems in burn survivors [14,15,16], as well as sexual problems which are largely unaddressed [17]. Among this summary of problems, the psychosocial impact of burn injuries can be considerable [18], severe enough to require mental health visits and targeted interventions [19].

Given the wide array of problems, the large differences between burn survivors, and the time constraints of healthcare providers, it can be challenging to identify care needs in the immediate or longer aftermath of burn injuries. Particularly during aftercare, attention to scar-related problems may dominate, and psychosocial problems may go unnoticed. However, a biomedical approach in which attention for burn survivors’ problems may be predominated by scar-related symptoms has been recognized as outmoded in favor of a biopsychosocial approach that takes into account a wider range of problems [20]. From a person-centered care perspective (PCC), identifying the full range of health problems is key in order to provide care that fits the burn survivor’s preferences and allows participation in decision-making. PCC is considered to fit the values, needs, and wishes of patients, which are discussed in a bilateral communication process [20].

In recent years, interest has grown in (early) screening following burns [21,22,23]. Particularly professionals working in the psychosocial field endorse screening in order to inform tailored interventions in a timely manner [18]. The use of a screening instrument may also be helpful during aftercare to grasp the full impact of problems with minimal time investment. In oncology, the cancer Distress Thermometer and problem list is a frequently used tool to identify a broad range of problems [24]. It was evaluated as a valuable tool to detect hidden distress and provide opportunities for discussion [25]. This tool was also tested on burn survivors [26], and although it was found valuable, it contained too many items irrelevant to burn survivors. This indicates that a burn-specific screening instrument should be used to meet burn survivors’ health problems.

One such instrument was developed in the United Kingdom by Gibson and colleagues [27]. The Adult Burn Patient Concerns Inventory comprises 58 items divided into several domains and aimed to improve communication and tailor patient-centered encounters [27]. Our aim was to develop a burn-specific screening instrument aligned with Gibson’s concern inventory. It started several years ago with a study in which we investigated the quality of life from the burn survivor’s perspective [28]. In our previous study, 99 items were identified as relevant aspects of quality of life from burn survivors’ perspectives. These items were derived from a focus group with six burn survivors, one-to-one interviews with 24 burn survivors, and a card-sorting task with 24 burn survivors. The items were divided into six health domains, including psychological well-being (subdomains: traumatic stress symptoms, cognitive symptoms, negative emotions, body perception, depressive symptoms), economic problems (subdomains: financial concerns and work), social well-being (subdomains: invalidation and stigmatization), physical well-being (subdomains: somatic symptoms, scars and functional limitations), sexuality and intimacy (subdomains: partner, anxiety/avoidance) and resilience (subdomains: positive coping and social sharing). However, further content refinement was deemed necessary to develop an easy-to-use screening instrument.

The current paper presents the development of a screening instrument for use in the outpatient setting for adult burn survivors. The instrument corroborates the PCC principles of screening problems from a biopsychosocial perspective, augmenting a therapeutic relationship, and considering the burn survivor’s values and sharing power and responsibility [20]. To test the face validity of the instrument, we hypothesized that physical problems (i.e., scar-related and daily functioning concerns) and psychological problems are more frequent concerns, in line with a review on quality of life [29]. We also explored whether the instrument would be able to detect time-related differences in concerns.

## 2. Materials and Methods

The starting point for the instrument’s content was the 99 items derived from a previous study from our research group [28] in which health-related quality of life (HRQL) was investigated from adult burn survivors’ perspectives. 

The first phase of this study comprised *content refinement* performed in two steps. First, staff with different backgrounds were recruited from two regional burn centers in the Netherlands to participate in the focus groups. The aim was to determine health domains relevant for screening during aftercare. Second, in an expert meeting consisting of aftercare nurses and nursing and psychological researchers, items were refined and reduced, checked for clarity, and rephrased when deemed necessary in order to determine the final item pool.

The second phase comprised the *cognitive evaluation* of the items. Participants were asked to review the wording of the items to ensure that the list was adequate, comprehensive, and comprehensible. Debriefings were conducted to assess the clarity and the consistent interpretation of the items. Each burn survivor provided feedback on the item set. The process of cognitive evaluation was performed using verbal probes, including asking patients to rephrase the items in their own words and pointing out words that were confusing or difficult to understand. 

In the third phase, the screening instrument was *tested* in outpatient settings. Burn survivors were invited to participate in the study when they visited the medical outpatient clinic of one of the two regional burn centers. They were asked to complete the instrument preceding their hospital appointment. They received the paper version by regular mail or by email. Some completed the instrument at the outpatient clinic when they had no time to complete it but were willing to do so in the waiting room or during the visit with the aftercare nurse. After the visit, they were asked their opinion about the completeness, comprehensibility of the items, and usefulness of the instrument. The answers were reviewed by the aftercare nurse. Oral information about the aim of the study was given and they were asked to give written consent or verbal assent in face-to-face contact with the researcher or aftercare nurse at the outpatient clinic. After giving consent, they were asked to provide the following information: gender, age, TBSA burned, whether they needed surgery and time postburn. This study was part of a broader evaluation of aftercare. A local review board approved the study (study number L2020031, 4 March 2020).

After the testing phase, one interview with a person (who did not have burns) with low literacy was conducted. The aim was to further simplify the wording and the clarity of the instructions provided to complete the instrument. Additionally, images illustrating the content of the health domains were added. The meaning of the images was tested in one male person with a non-Western background with burns by asking what he thought the images represented.

All statistical analyses were conducted with IBM SPSS version 27. Frequencies of the items were inspected to see whether items were redundant. Because the different health domains had an unequal number of items, a mean score of the items of each domain was calculated. Pearson correlations were calculated between the health domains and the Distress Thermometer (DT). To test the face validity of the instrument, the mean domain scores were entered into a linear regression analysis predicting the DT. Backward deletion was used with the aim of retaining health domains associated with higher levels of distress in order to check concordance with the extant literature. Student *t*-tests were used to compare means between burn survivors who sustained burns less and more than 6 months postburn.

## 3. Results

### 3.1. Phase 1: Content Refinement

Two focus groups were conducted in the burn centers of Rotterdam and Beverwijk, the Netherlands, in September and October 2018, respectively. Fifteen staff members with the following backgrounds attended the focus groups: psychologists, social workers, physiotherapists, aftercare nurses, chaplain/spiritual counselors, (research) nursing and psychological researchers, medical doctors, and occupational therapists. The domains that were indicated relevant to screening included physical and functional well-being, psychological well-being and cognitive functioning, social well-being, spiritual well-being, intimacy concerns, financial concerns, work-related issues, and positive coping. In fact, staff members agreed with the initial cluster identification of the burn survivors’ perspectives as presented in Kool et al. [28], and consequently, the starting point for the next phase was the 99 items underlying the clusters.

In an expert meeting with aftercare nurses and researchers, these domains were further inspected and critically appraised. The final selection of items was performed in several steps. First, the previously described 99 items resulting from burn survivors’ perspectives [28] were appraised regarding the usefulness for the screening tool. Several items related to the same topic (for example being tired easily and having low energy both relate to fatigue) were grouped together. Second, (sub)domains were further refined, and the allocation of items to subdomains was discussed. In the last step, the aftercare nurses critically evaluated the wording. The following health domains were determined: ‘scars’, ‘daily life functioning’, ‘scar treatment’, ‘body perceptions’, ‘stigmatization’, ‘intimacy’, ‘mental health’, ‘relationships with other people’, ‘financial concerns’, and ‘positive coping’. All health domains identified in the focus groups were maintained. However, spiritual well-being was not included as a separate domain, but one item relating to meaning giving was included in the mental health domain. Furthermore, a Distress Thermometer ranging from 0 (not affected) to 10 (extremely affected) was added to indicate the extent to which the problems caused distress, and an item inquiring about the need to discuss their concerns with a healthcare provider was included.

### 3.2. Phase 2: Cognitive Evaluations

Four adult burn survivors reviewed the wording of the items and appraised the completeness of the item pool. One person was a member of a patient panel and was interviewed outside the hospital. Three burn survivors were recruited at the outpatient clinic and were subsequently interviewed. There was a mixture of male and female burn survivors, and they ranged in age between 30 and 55 years old. One person suggested adding camouflage techniques as an item that was deemed relevant. It was added to the scar treatment domain. Several items were reworded to improve clarity.

### 3.3. Phase 3: Testing the Instrument

A total of 102 adult outpatients completed the screening instrument preceding an outpatient visit. The mean age was 43.8 (SD = 18; range 18–88), 64 were male (62.7%), the mean TBSA burned was 6.4% (SD = 8; range 0.5–45), and 58 (57%) needed surgery. The mean time postburn was 8.1 months (SD = 11.2; range 0.5–76). 

The self-reported DT was, on average, 3.9 (SD = 2.7), ranging from 0 (indicated by 13% of the burn survivors) to 10 (2% of the burn survivors). Table 1 presents the frequencies of the items indicating that all items were scored, ranging from 5% (items: problems with cuddling or insurance) to 61% (item: support from others). When leaving aside the positive coping items, the most prevalent were concerns related to scars (81% scored at least one item) and mental health (70% scored at least one item).

**Figure 1:**
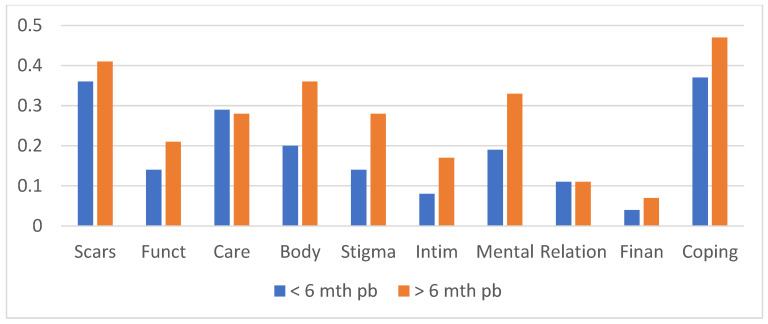
Means of the health domains in burns survivors <6 months postburn (blue bars) and > 6 months postburn (orange bars). Bars represent the means of the respective health domains: scars, functioning, care, body perceptions, stigma, intimacy, mental health, relations, financial concerns, and coping.

To explore whether the screening instrument would be able to detect differences across time, burn survivors who sustained their injury less than 6 months earlier (N = 61; 60%) were compared to those with longer time postburn (N = 41; 40%). Figure 1 shows that the subgroup > 6 months postburn had statistically significantly higher mean domain scores relating to ‘body perceptions’ (*p* = 0.03), ‘stigmatization’ (*p* = 0.006), ‘mental health’ (*p* = 0.004). Functioning (*p* = 0.07) and intimacy (*p* = 0.06) were borderline significant.

Table 1 presents the Median DT per item. Median DT for checked items ranged between 5 and 7.5 (except for scar treatment and relations), whereas Median DT for unchecked items ranged from 2 to 4. This may indicate that a DT ≥ 5 calls for clinical attention.

**Figure 2:**
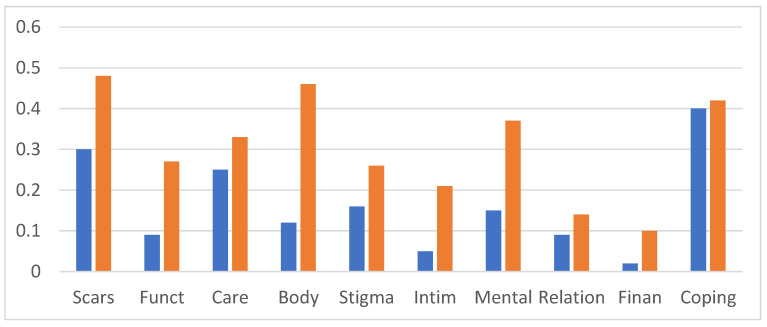
Means of the health domains in burns survivors scoring <5 (blue bars) and ≥5 DT (orange bars). Bars represent the means of the respective health domains: scars, functioning, care, body perceptions, stigma, intimacy, mental health, relations, financial concerns, and coping.

Figure 2 illustrates the mean scores for the health domains for those with DT < 5 (N = 58; 57%) and DT ≥ 5 (N = 44; 43%). It shows substantial differences in all health domains except for ‘scar treatment’. Medians (Table 1) may also provide insight into which items were associated with higher distress. For example, with regards to the health domain ‘scars’, it showed that pain, restrictive and stiff scars were associated with the highest distress. In the ‘mental health’ domain, particularly concentration and sleep problems were related to higher DT. 

**Figure 3:**
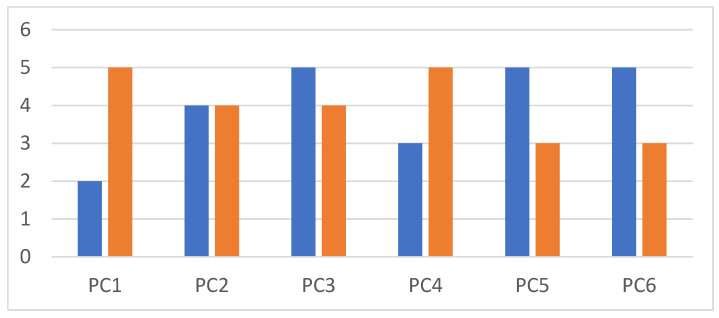
Median DT per item for burn survivors who left the item unchecked the item (blue bars) and who checked the item (orange bars). Positive coping PC1 = social support, PC2 = focusing on the positive, PC3 = enjoying the little things, PC4 = standing up for yourself, PC5 = remembering that things could be worse, PC6 = humor.

The items included in the ‘positive coping’ domain showed that, particularly, ‘social support’ (*p* = 0.076) and ‘standing up for yourself’ (not statistically significant) were related to higher DT, whereas the other coping strategies showed to be related to lower DT, indicating that in times of distress, particularly ‘social support’ may be important. Figure 3 visually illustrates this.

To test whether the domains were interrelated and related to the DT, Pearson correlations were calculated. As shown in Table 2, most health domains showed significant correlations with the DT, excluding ‘scar treatment’ and ‘positive coping’. This indicates that ‘scar treatment’ and ‘positive coping’ did not have a direct relationship with distress. Furthermore, ‘positive coping’ significantly correlated with concerns related to ‘scars’, ‘body perceptions’, and ‘mental health’, indicating that more concerns were associated with higher levels of coping.

In the linear regression analyses with backward deletion, five domains were deleted in five steps: ‘intimacy’, ‘relations’, ‘financial concerns’, ‘scar treatment’, and ‘stigmatization’. In the last step, five domains were retained: ‘scars’, ‘daily life functioning’, ‘body perceptions’, ‘mental health’, and ‘positive coping’ (F(5,96) = 23.423, *p* < 0.0001) as presented in Table 3. The four problem domains showed a positive association with the DT, whereas positive coping showed a negative association with the DT. The final model explained 53% of the model variance.

Thirty-four persons (34%) preferred to discuss their concerns with a healthcare provider. They had a significantly higher DT (M = 5.94; SD = 2.16) compared to those who did not want to talk about it (M = 2.96; SD = 2.40), t(df = 99) = 6.102, *p* < 0.001. Consequently, the majority (N = 64) was not referred; thirteen had a short (<15 min) additional conversation with an aftercare nurse, twenty-two persons were referred to the aftercare nurse, two persons were referred to the psychologist, and one person was referred to social work.

### 3.4. Qualitative Results

Participants were asked their opinions about the screening instrument. Regarding completeness, most found that a sufficiently broad range of topics was addressed, and they found it relevant to indicate both physical and psychosocial problems. Regarding comprehensibility, many reported that the items were easy to understand and took limited time to complete, varying between 3 and 5 min. However, there were also reports that not all items were easy to comprehend. Regarding usefulness, it was stated it helped them to prepare for the conversation with the medical doctor. Seeing all those concerns at a glance provoked the response that it was confronting in a minority of the participants (12 out of 75 persons (16%)), whereas others stated it helped them to put things into perspective and to reflect on the situation.

### 3.5. Adjustments after the Testing Phase

Given the qualitative findings that items and instructions were not clear to every burn survivor, the wording of the items and instructions needed adjustment. An interview with a person with low literacy led to numerous changes (>20) in the wording and test instructions, and the suggestion to add illustrations. Most changes included replacement by easier-to-understand synonyms; for example, ‘fatigue’ was replaced by ‘tiredness’, ‘interference with daily life’ was changed into ‘problems in daily life’, and some instructions were adjusted or refined. Some items comprising several aspects were split. For example, in the mental health domain, the item comprising guilt, shame, and anger was split into two items: ‘guilt and shame’ (i.e., self-conscious emotions) and ‘anger’ (i.e., basic emotion). The item ‘concentration and memory’ was also split into separate items. These changes explain why the final version presented in Appendix A deviates from the tested version, as presented in Table 1.

Subsequently, for every health domain a representative image was added to illustrate the core meaning of the domain. The burn survivor who was asked to evaluate the images indicated the core domains by simply seeing the images. The final Dutch version, including the suggestions of the person with low literacy, was translated into English and is presented in the Appendix A. It should be noted that the English version was not checked on the level of literacy.

## 4. Discussion

This study describes the development of a brief screening instrument for a broad range of physical and psychosocial problems in the aftermath of a burn event. Based on 99 items from a previous study [28], two focus groups with healthcare professionals and an expert meeting with nursing and psychological professionals resulted in the Aftercare Problem List. It comprises 43 items divided into nine health domains screening specific concerns after burns and one domain including six items indexing positive coping strategies. Every health domain is illustrated with images representing the health domain.

The nine health domains included in the Aftercare Problem List considerably overlap with the domains included in the Adult Burns Patient Concern Inventory [27], i.e., concerns related to scars, physical problems, body image, treatment, mental health, and social well-being. These health domains are established long-term problems affecting the quality of life in the burns population [23,29], emphasizing their relevance in the aftermath of burns. 

Although the instrument was not developed for diagnostic purposes, face validity was tested by relating the health domains to the DT. In a regression analysis, five health domains were retained that were associated with distress, including ‘scars’, ‘daily life functioning’, ‘body perceptions’, ‘mental health’, and absence of ‘positive coping’. Additionally, our screening instrument comprises a Distress Thermometer that was useful in differentiating (≥5) between burn survivors with low and high distress. The cutoff point of five on the DT was also suggested in the oncology literature to be indicative of significant distress [30,31] as well as in an earlier study from our group using that screening list in burn survivors [26]. Furthermore, the Aftercare Problem List showed that burn survivors who sustained their injury more than 6 months ago had higher concerns relating to ‘body perceptions’, ‘stigmatization’, and ‘mental health’. This finding corroborates studies that indicate the long-term impact on psychosocial functioning after sustaining burns [32]. It also emphasizes the relevance of screening for this type of problem, particularly in burn survivors who visit the outpatient clinic months to years after their burn injury.

Our screening instrument also includes positive coping. Pearson correlations showed a positive association between ‘positive coping’ and more concerns in the health domains ‘scars’, ‘body perceptions’, and ‘mental health’. On a single-item level, particularly ‘social support’ was shown to be related to higher DT. Although this may be counter-intuitive, it indicates that coping strategies are more often consciously relied upon when burn survivors experience more concerns, demonstrating that in distressing times, there is, for example, a greater need for social support. This corroborates findings from a qualitative study that reassurance from social support (such as indicating that the burn survivor was still attractive or the same person) was important to strengthening body image [33]. In addition, the results of the linear regression analysis indicate that, when controlling for health problems, the absence of positive coping is related to higher distress. Therefore, high distress in the absence of positive coping strategies may be a point of attention for healthcare workers. The role of social support in successful rehabilitation, be it support from significant others or peers, is well-established [34,35]. When patients report problems and a lack of positive coping, healthcare providers may recommend referral to peer support groups in order to facilitate rehabilitation.

This study subscribes to the usefulness of single items for screening purposes, in line with another empirical report [36]. It was unsurprising to find physical (e.g., scars-related problems) and mental health concerns to be associated with distress as these domains were also identified in a review (e.g., [29]) to affect quality of life. The study findings were surprisingly comparable to outcomes measured with multi-item measures but warrant further testing to determine exactly how they relate to multi-item measures.

This study has some implications for clinical practice. First, the Aftercare Problem List, comprising both physical and psychosocial items, can be used for screening purposes, which is in line with recommendations emphasizing the need for screening problems after burns [22]. As it uncovers psychosocial problems and inquires about preference to discuss these concerns, it helps to increase psychological presence and may reduce stigma related to mental health problems; both were recently identified as barriers for burn survivors to seek psychological help [37]. Second, the screening may assist in selecting suitable (psychosocial) interventions. Although psychological intervention studies are limited in the burns field, there are studies available that indicated the usefulness of different therapies in this population. For example, Acceptance Commitment Therapy (ACT) [38] and Cognitive Behavioral Therapy [39] may be an appropriate therapy for body image-related problems, depressive and posttraumatic stress symptoms, and referral to peer groups to increase social support and social sharing [34]. Sometimes, a supportive conversation about usually hidden problems (for example, relational difficulties) with the aftercare nurse can be an adequate answer to relieve the burn survivor’s burden. Third, it may be challenging to implement a screening instrument in clinical practice due to limited opportunity and willingness of the staff because it requires changes in work routines.

Strengths of the screening list include the single items that are easy to understand and require limited time to complete, increasing the usefulness for a large group of burn survivors. The addition of images may further broaden the applicability of the instrument in burn survivors with low language proficiency and may facilitate the identification of their needs. This may diminish health disparities and provide high-quality care to all patients with burns. The images and DT distinguishes this instrument from the Adult Burn Patient Concerns Inventory [27]. This study has some limitations. First, the allocation of items into health domains was not statistically tested. Second, the wording of the items was appraised by a person with low literacy, but this was performed after the testing phase and only by one person, while two or three would have been preferable. The insight to perform this resulted from the test phase that revealed difficulties with the wording in some burn survivors, despite the cognitive testing in burn survivors. However, patient panels usually do not comprise persons with low literacy, and therefore, we recommend including testing on low literacy as a standard procedure in the development of a new instrument.

Further research should investigate the adjusted version and may specifically investigate the usefulness of the instrument with images in patients with limited language proficiency. It would also be interesting to investigate how the instrument with several single items relates to validated questionnaires or diagnostic instruments. If such a relationship were established, it could be used as a stepped-screening method, meaning that when the patient scores positive on a single item, only then a more in-depth (diagnostic) screening may follow. Future research may also investigate the optimal timing of applying the screening, the optimal way of administration (in advance or at the outpatient clinic) and patient engagement with multiple administrations. However, the results of this study indicated that the instrument seems to be useful in burn survivors who visit the outpatient clinic beyond the subacute phase because particularly this group showed more problems in the psychological domain that may call for further attention.

## 5. Conclusions

In summation, this study showed that a fast and easy screening instrument could yield important information about burn survivors’ concerns after burns. Health domains expected to dominate in burn survivors were indeed found to be significantly related to distress. The instrument also supports principles of PCC, such as its biopsychosocial perspective, and it may assist in sharing power and responsibility between survivors and healthcare staff as it helps the burn survivor prepare for the medical outpatient visit and leaves the responsibility to talk about the concerns with the burn survivor.

## Figures and Tables

**Table 1:** Frequency and Median of the items and number of items checked and means per domain.

Health Domain	N (%)	N (%) ≥ 1 Item Checked	Mdn DT Item Checked/ Mdn DT Item Unchecked
**1 Scars**		83 (81.4)	
Pain	34 (33.3%)		6/2.5
Itch	56 (54.9%)		5/2.5
Tightening of scars that restrict movement	48 (47.1%)		6/2
Stiffness of scars	36 (35.3%)		6/3
Feeling hot and can’t cool down	8 (7.8%)		5/3
Skin discoloration	41 (51.2%)		5/4
**2 Daily life functioning**		60 (58.8)	
Using your hands	21 (20.6)		5/3
Cycling or climbing stairs	8 (7.8)		5/4
Standing still for a long time	16 (15.7)		5/3
Being dependent on others	19 (18.6)		7/3
Having less contact with others	19 (18.6)		7/4
Tiredness	31 (30.4)		5/3
**3 Scar treatment**			
Rubbing lotion onto your scars	51 (50)		4/4
Fragile skin that tears easily	14 (13.7)		6/3
Wearing pressure garments	30 (29.4)		6/3
Silicone or camouflage treatment	12 (15.8)		6.5/3.5
**4 Body perceptions**		47 (46.1)	
Feeling insecure about your body because of the scars	26 (25.5)		5.5/3
Wanting to cover up your scars	30 (29.4)		6/3
Finding yourself less attractive	25 (24.5)		7/3
**5 Stigmatization**		46 (45.1)	
Remarks or questions	30 (29.4)		5.5/3
Staring	21 (20.6)		7/3
Lack of understanding	10 (9.8)		6/3.5
**6 Intimacy**		26 (25.5)	
Revealing your scars	19 (18.6)		6/3
Someone touching your scars	17 (16.7)		7/3
Cuddling	5 (4.9)		6.5/4
Sexual intercourse	7 (6.9)		6.5/4
**7 Mental health**		71 (69.6)	
Reliving the accident	18 (17.6)		5/3
Heightened awareness of danger	31 (30.4)		5/3
Feeling guilty, ashamed or angry	25 (24.5)		5/3
Feeling depressed	29 (28.4)		5/2.5
Sleeping badly	21 (20.6)		7.5/3
Difficulty concentrating and poor memory	28 (27.5)		7/3
Wondering why the accident happened	23 (22.5)		5/3
**8 Relationships**		25 (24.5)	
Within the family unit	12 (11.8)		3/4
Within the extended family	12 (11.8)		2.5/4.5
With friends	11 (10.8)		4.5/4
At work or school	10 (9.8)		6/3
**9 Financial concerns**		9 (8.8)	
With financial matters	6 (5.9)		6.5/3.5
With insurance	5 (4.9)		7/4
**10 Positive coping**		90 (88.2)	
Support from family and friends	62 (60.8)		5/2
Focusing on the positive	45 (44.1)		4/4
Enjoying the little things	37 (36.3)		4/5
Standing up for yourself	50 (49.0)		5/3
Remembering that things could always be worse	19 (18.6)		3/5
Humor	39 (38.2)		3/5

Note. Silicone or camouflage treatment had 26 missing values because it was added during the evaluation process.

**Table 2:** Pearson correlations.

	DT	D1	D2	D3	D4	D5	D6	D7	D8	D9	
DT	-										
D1 Scar	0.47 **	-									
D2 Functioning	0.43 **	0.33 **	-								
D3 Treatment	0.19	0.26 **	0.13	-							
D4 Body	0.60 **	0.39 **	0.38 **	0.20 *	-						
D5 Stigma	0.30 **	0.28 **	0.38 **	0.25 *	0.48 **	-					
D6 Intimacy	0.44 **	0.26 **	0.40 **	0.10	0.62 **	0.37 **	-				
D7 Mental	0.62 **	0.37 **	0.51 **	0.08	0.68 **	0.38 **	0.56 **	-			
D8 Relations	0.23 *	0.16	0.31 **	0.20 *	0.21 *	0.16	0.20 *	0.30 **	-		
D9 Financial	0.28 **	0.09	0.34 **	−0.01	0.21 *	0.19	0.28 **	0.33 **	0.27 **	-	
D10 Coping	0.09	0.26 **	0.19	0.06	0.24 *	0.14	0.15	0.35 **	0.07	0.08	
Mean	3.6	0.38	0.17	0.29	0.26	0.20	0.10	0.25	0.11	0.05	0.41
SD	2.7	0.26	0.20	0.24	0.35	0.26	0.24	0.25	0.22	0.18	0.29

Note. DT = Distress Thermometer, D = Domain. * indicates *p* < 0.05; ** indicates *p* < 0.01.

**Table 3:** Linear regression analysis predicting DT.

Variable	B	SE	Beta	t	*p*-Value
Constant	1.86	0.39		4.74	0.000
Scars	2.31	0.81	0.22	2.85	0.005
Daily life functioning	3.21	1.11	0.23	2.89	0.005
Body perceptions	2.02	0.74	0.26	2.74	0.007
Mental health	3.32	1.14	0.30	2.92	0.004
Positive coping	−1.70	0.70	−0.18	−2.41	0.018

## Data Availability

The participants of this study did not give written informed consent for their data to be shared publicly, so due to the small sample and sensitive nature, data are not available.
